# Impact of COVID-19 infection on pregnancy outcomes and the risk of maternal-to-neonatal intrapartum transmission of COVID-19 during natural birth

**DOI:** 10.1017/ice.2020.84

**Published:** 2020-03-19

**Authors:** Suliman Khan, Liangyu Peng, Rabeea Siddique, Ghulam Nabi, Nawsherwan, Mengzhou Xue, Jianbo Liu, Guang Han

**Affiliations:** 1 Department of Cerebrovascular Diseases, The Second Affiliated Hospital of Zhengzhou University, Zhengzhou, China; 2 Department of Gynecology and Obstetrics, Renmin Hospital of Wuhan University, Wuhan, Hubei Province, China; 3 Key Laboratory of Animal Physiology, Biochemistry and Molecular Biology of Hebei Province, College of Life Sciences, Hebei Normal University, Shijiazhuang, China; 4 Department of Public Health, Wuhan University, Wuhan, China; 5 Department of Respiratory Diseases, The Second Affiliated Hospital of Zhengzhou University, Zhengzhou, China; 6 Department of Radiation Oncology, Hubei Cancer Hospital, Tongji Medical College, Huazhong University of Science and Technology, Wuhan, China

*To the Editor—*Atypical pneumonia known as coronavirus disease (COVID-19), which is caused by the SARS-CoV-2 virus, is highly infectious and is currently spreading rapidly around the globe. Since the emergence of SARS-CoV-2 in Wuhan, Hubei Province, China, during December 2019, it has caused thousands of morbidities and mortalities around the globe.^1^ Many studies have focused on infected patients from the general population; however, details related to pregnancy outcomes of women with COVID-19 are scarce. Chen et al^2^ reported the maternal–neonatal outcomes and vertical transmission potential of COVID-19 pneumonia in pregnant women. Their report focused on pregnant women who delivered babies through C-section only, and no case for normal vaginal delivery has been reported. Moreover, healthcare workers were not included, even though healthcare workers are at higher risk of contracting the infection and psychological consequences.^3^ We conducted a case report study in pregnant women with laboratory-confirmed SARS-CoV-2 with vaginal deliveries at Renmin Hospital, Wuhan, China. Both healthcare workers and female obstetric patients were included in this study.

Here, we present a case report study on pregnant women (n = 3) infected with COVID-19 admitted to Renmin Hospital between January 28 and March 1, 2020. COVID-19 pneumonia was diagnosed according to the new Coronavirus Pneumonia Prevention and Control Program (4th edition) published by the National Health Commission of China (Fig. 1, Table 1).^2,4^ All 3 pregnant women were positive for SARS-CoV-2 using quantitative reverse-transcriptase polymerase chain reaction (qRT-PCR) on specimens from the respiratory tract (nasal and pharyngeal swabs) and blood specimens. To determine neonatal infection with COVID-19, cord blood and neonatal throat swab samples were collected within 12 hours after delivery in the operating room and were tested using qRT-PCR. All available data are presented in Table 1 as they relate to maternal-neonatal outcomes. The study protocol was approved by the ethical review board of the Renmin hospital.

**Table 1.** A Summary of Maternal and Neonatal Outcomes Infected With COVID-19

Case No.	1	2	3	n/N (%)
Maternal and neonatal outcomes				
Age, y	28	33	27	…
Gravida (G) and para (P)	G1, P1	G1, P1	G1, P1	…
Gestational age on admission, weeks^+days^	34^+6^	39^+1^	38^+2^	…
Mode of delivery				
Vaginal route	Yes	Yes	Yes	3/3 (100)
Sign and symptoms				
Fever	Yes	Yes	No	2/3 (66.6)
Cough	Yes	Yes	Yes	3/3 (100)
Chest tightness	No	No	Yes	1/3 (33.3)
Infected with SARS-Cov-2	Yes	Yes	Yes	3/3 (100)
Laboratory results				
White blood cell count (×10^9^ cells per L)	7.5	11.7	12.9	…
Lymphocyte count (×10^9^ cells per L)	1.1	2.76	1.06	…
C-reactive protein (mg/L)	32	18.4	1.5	…
ALT (U/L)	10	16	6	…
AST (U/L)	30	27	13	…
Neonatal outcomes				…
Apgar score, 1 min–5 min	8–9	9–10	9–10	…
Birth weight, g	2,890	3,500	3,730	…
Birth length, cm	48	50	51	…
Preterm delivery	Yes	No	No	1/3 (33.3)
Neonatal death/still birth	No	No	No	0/3 (0.00)
Infected with SARS-Cov-2	No	No	No	0/3 (0.00)

Note. ALT, alanine aminotransferase; AST, aspartate aminotransferase.

## Case 1

Patient 1 was a 28-year-old woman (gravida 1, para 1) at 34 weeks plus 6 days gestation. On admission (January 28, 2020), she presented with the onset of fever and cough. She had a history of contact with a person with COVID-19, and her body temperature was 37.3°C. The laboratory test of the nasopharyngeal swab was positive for SARS-CoV-2. On January 28, at 34 weeks plus 6 days of pregnancy, a live preterm baby was delivered vaginally. The newborn responded well, with Apgar scores of 8 and 9 at 1 minute and 5 minutes, respectively. The neonatal birth weight was 2,890 g and the birth length was 48 cm. The newborn tested negative for SARS-CoV-2 and remained under observation in the neonatal department. To prevent and control postpartum infection, the patient was given an intravenous injection of azithromycin, oral Lianhua Qingwen capsules (Chinese medicine), and oseltamivir antiviral drugs.

## Case 2

Patient 2 was a 33-year-old woman (gravida 1, para 1) at 39 weeks plus 1 day gestation. She was admitted to the hospital on February 22, 2020, with the onset of fever and cough. Her body temperature was 37.6°C. She had a history of contact with a family member who had COVID-19. The laboratory test of a nasopharyngeal swab was positive for SARS-CoV-2. On February 22, at 39 weeks plus 1 day of pregnancy, she had a vaginal delivery. The baby had an Apgar score of 9–10, a birth weight of 3,500 g, and a birth length of 50 cm. The newborn tested negative for SARS-CoV-2. The patient was given antibiotics, antiviral drugs, and intermittent oxygen inhalation.

## Case 3

Patient 3 was a 27-year-old woman (gravida 1, para 1) at 38 weeks plus 2 days gestation. She was admitted to the hospital on March 1, 2020, with the onset of cough and chest tightness. She had a history of contact with a person with COVID-19. The laboratory test of the nasopharyngeal swab was positive for SARS-CoV-2. The fetal heartbeat was good and the ultrasound examination was normal. On March 1, at 38 weeks plus 2 days of pregnancy, she vaginally delivered a full-term live baby with an Apgar score of 9–10, a birth weight of 3,730 g, and a birth length of 51 cm. The baby laboratory test of the nasopharyngeal swab was negative for SARS-CoV-2. The baby was transferred to the pediatric ward and remained under observation. The patient was given antibiotics, antiviral drugs, Chinese medicine, and intermittent oxygen inhalation.

## Discussion

**Fig. 1.**
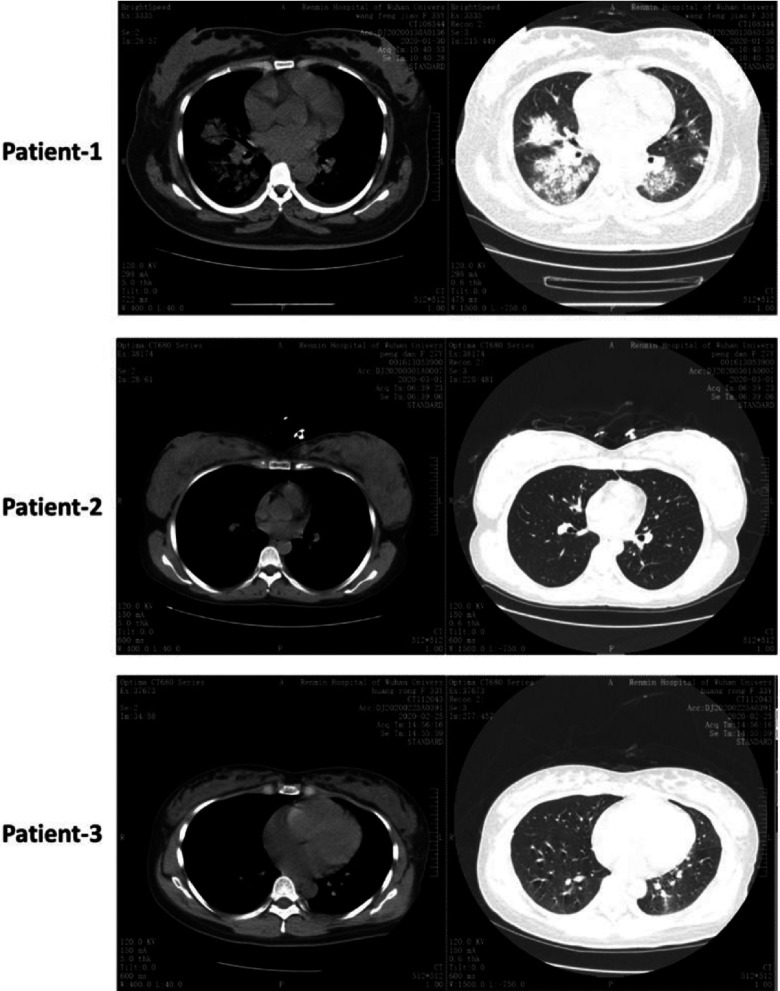
Computed tomography (CT) chest scans of 3 patients. This figure shows patchy consolidation and opacities.

We report a case report study of 3 pregnant women with laboratory-confirmed COVID-19 pneumonia (Fig. 1, Table 1). All 3 pregnant women had vaginal deliveries. These patients presented with symptoms manifested by people with COVID-19.^2^ Of 3 patients, only 1 patient (case 1) delivered a preterm baby. However, the preterm baby (case 1) tested negative for SARS-CoV-2, which suggests that the preterm delivery was not caused by vertical transmission of SARS-CoV-2. However, the preterm delivery may have been caused by psychological stress during pregnancy associated with COVID-19 pneumonia. Chen et al^2^ reported that 4 of 9 patients had preterm delivery but that preterm delivery was not associated with COVID-19 pneumonia. However, they believed that preterm delivery was associated with severe preeclampsia and other complications that were not observed in our study.

We did not observe neonatal death or stillbirth in the 3 patients included in our report. However, during 2002–2003 pandemic, a study was conducted that included 12 pregnant women infected with SARS-CoV.^4^ In that study, 4 of 7 pregnant women (57%) had a miscarriage in the first trimester of pregnancy and 4 of 5 pregnant women (80%) had preterm delivery.^4^ Also, 2 of 3 patients (66.6%) had elevated C-reactive protein (>10 mg/L).^4^ Like Chen et al,^2^ we also found elevated C-reactive protein (>10 mg/L) in pregnant women with COVID-19 pneumonia. In our study, the neonatal birth weights ranged from 2,890 g to 3,730 g and the neonatal birth lengths ranged from 48 cm to 51 cm. All 3 neonates had normal Apgar scores ranging from 8 to 10 at 1 minute and at 5 minutes after birth. All neonates tested negative for SARS-CoV-2.

This case report study was limited by a small sample size. A study with a larger sample size should be encouraged to investigate the possibility of COVID-19 vertical transmission in the second and third trimesters of pregnancy and possible adverse pregnancy outcomes. In summary, none of the 3 women in this study had died of COVID-19 infection as of March 1, 2020. No vertical transmission of COVID-19 was found in the third trimester of pregnancy among infants delivered via the vaginal route. Moreover, we did not find evidence of maternal-to-neonatal intrapartum transmission of COVID-19 via vaginal delivery.
